# Automatic Delineation of Tumor Spheroids in Microscopic Images Using Deep-Learning

**DOI:** 10.1021/acsmeasuresciau.5c00172

**Published:** 2026-02-02

**Authors:** Jens Maus, Janina Nitschke, Pavel Nikulin, Frank Hofheinz, Mareike Barth, Sandy Lemm, Lena Richter, Jens Pietzsch, Anja Braune, Martin Ullrich

**Affiliations:** † Institute of Radiopharmaceutical Cancer Research, 28414 Helmholtz-Zentrum Dresden-Rossendorf, 01328 Dresden, Germany; ‡ Faculty of Chemistry and Food Chemistry, School of Science, Technische Universität Dresden, 01069 Dresden, Germany; § Department of Nuclear Medicine, University Hospital Carl Gustav Carus, Technische Universität Dresden, 01307 Dresden, Germany; ∥ Faculty of Medicine and University Hospital Carl Gustav Carus, Technische Universität Dresden, 01307 Dresden, Germany

**Keywords:** Tumor Spheroid Imaging, Radiopharmacological Treatment Response Assays, Delineation, Cancer, Deep-Learning, Artifical Intelligence, Convolutional Neural Networks

## Abstract

Tumor spheroid growth assays are used to evaluate the potential of cancer therapies in vitro. During such experiments, extensive microscopic image series are generated, which are commonly analyzed using threshold-based delineations. However, due to treatment-induced morphological changes of the spheroids, very time-consuming manual corrections are often required. The goal of our work was the development of an AI-based method for accurate and automated delineation of spheroid growth assays, ultimately reducing the reliance on manual delineation and corrections. Spheroids were grown from mouse pheochromocytoma (MPC) cells and subjected to irradiation with particle-emitting radioligands. Spheroid growth was monitored over 35 days. N = 38090 images, acquired within seven experiments and two studies, were included. Spheroids were delineated with a threshold-based method followed by manual corrections and the resulting delineations served as ground truth for network training and testing. The data were divided into two independent data sets: one for training and internal validation using a 5-fold cross-validation (N = 21567; main data set) and another for final independent testing (N = 16523). The network was developed using the nnU-Net v2 deep-learning (DL) framework. DL-based and manual delineations were compared using the *Dice similarity coefficient* (DSC). Additionally, treatment effects in a spheroid experiment were compared by quantifying half-maximum spheroid control doses (SCD_50_). The median DSC values in the main and test data sets were 0.979 and 0.974, respectively. In the main data set, only 7% (N = 1571) of the DL-generated delineations and 8% (N = 1304) in the test data set showed DSC < 0.9, indicating high performance. The SCD_50_ values were comparable between manual (day 13: 0.086 ± 0.001, day 35: 0.150 ± 0.001) and DL-based delineations (day 13: 0.083 ± 0.002, day 35: 0.149 ± 0.007). The network enables fast and accurate delineation of tumor spheroids in treatment response assays, reducing the time needed to delineate all spheroid images of a single experiment from several days with the previously applied method to a few hours only.

## Graphical abstract

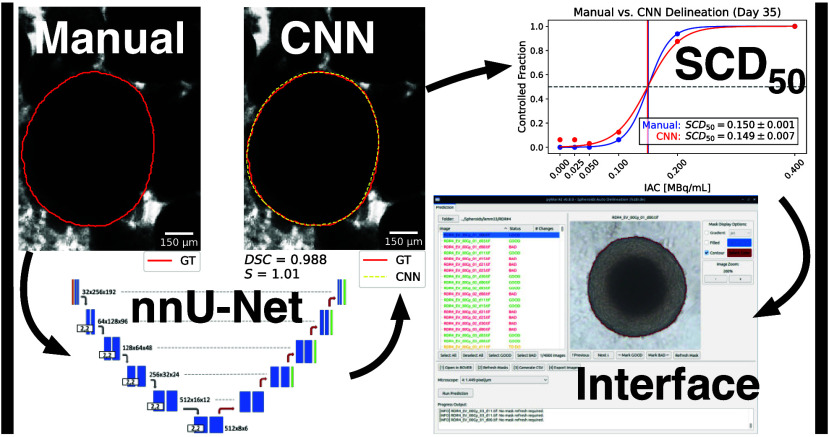

## Introduction

Tumor spheroid growth response assays are commonly used in cancer research to evaluate the potential of new and existing cancer therapies in vitro. These assays allow researchers to determine the susceptibility of specific solid tumor entities to antineoplastic treatments including, among others, pharmacotherapies (PT), external beam radiation therapies (EBRT), radionuclide therapies (RNT), and combinations thereof, at an early stage before in vivo studies are conducted.^1^ Tumor spheroids, as three-dimensional models, offer a valuable opportunity to study cancer treatments while taking tumor tissue characteristics into account that are not present in monolayer cultures, but known to modulate therapeutic efficacy.^1^ These characteristics include restricted perfusion, hypoxic and necrotic regions, altered interstitial pressure, genetic and phenotypic heterogeneity, nutrient supply gradients and barriers, as well as cell–cell and cell-matrix communications.^2,3^ In addition, spheroids formed from two or more cell types prove to be a suitable model for studying tumor stroma interactions and cellular signaling.^2,4^ Hundreds of spheroids are typically imaged microscopically for several weeks during one single experiment, resulting in large image series used for subsequent growth analysis.

At the beginning of treatment, the distinct spherical shape of most tumor spheroids is typically suitable for straightforward threshold-based delineations to measure their size. However, morphologic responses of tumor spheroids to antineoplastic treatments are sometimes not only associated with changes in spheroid size, but can also lead to severe morphological disintegration. Consequently, large amounts of cellular debris can accumulate in the culture dish, hindering the threshold-based delineation of the surviving spheroid remnants. As a result, spheroid remnants embedded in high-contrast debris must be manually delineated by experienced observers, which is partly error-prone and extremely time-consuming. Therefore, the development of alternative methods to automate the delineation of spheroids are required.

Over the past years, deep-learning (DL) with neural networks in general and *Convolutional Neural Networks* (CNNs) in particular have emerged as a pivotal data processing technique in the biomedical imaging field.^5^ They demonstrated their effectiveness in various segmentation tasks, including delineation of structures obtained via microscopic imaging. In tumor spheroid imaging, other research groups have already demonstrated the effectiveness of CNNs. Sadanandan et al.^6^ employed a U-Net-like network^7^ to successfully delineate spheroids across a diverse range of cell types, treatments, and experimental conditions. Lacalle et al.^8^ demonstrated that an HRNet-based model^9^ can outperform a standard U-Net for spheroid segmentation. However, their study focused on generalization performance of different models across three different microscopes, while their data set size was relatively low with only 838 spheroid images being used for the network training phase. Additionally, the spheroid images analyzed in both of the mentioned studies lacked extensive amounts of debris, which, if present like in our data set can considerably complicate a delineation task.

Recently, however, Streller et al.^10^ showed that a U-Net can still outperform a HRNet when tuned carefully. They have also demonstrated that this statement holds true even for spheroid images with debris or fragmentation in place. Requirement for the extensive manual tuning of the network architecture and the training process makes it, however, difficult to follow this approach to develop new CNN models for further cell and therapy types which might lead to varying spheroid morphologies and different debris/fragmentation levels. The deep-learning based nnU-Net^11^ framework which is widely used in the biomedical imaging field promises to address this problem. nnU-Net automatically derives necessary hyperparameter values for the network training using a set of heuristic rules adapting to the specific properties of the training data automatically. It is, however, unknown so far how its self-configuration properties work with microscopic spheroid images.

In our study, we evaluated the accuracy of spheroid segmentation using an nnU-Net-based network trained on manually delineated mouse pheochromocytoma (MPC) tumor spheroids exposed to beta-minus particle-emitting radionuclides. We compared the segmentation of spheroids in a large data set comprising several tens of thousands of spheroids at different progression stages extracted from seven ongoing experiments and two published studies.^3,12^ The segmentation performance was evaluated based on treatment-associated changes in spheroid size, and treatment efficacy calculated as the half-maximum spheroid control dose (SCD_50_) metric, which corresponds to the initial activity concentration (IAC) required to control 50% of the spheroids for a defined period of time.

## Material and Methods

### Spheroid Preparation and Imaging

Tumor spheroids were generated from an initial number of 1000 MPC cells seeded into 96-well concave-bottom ultralow attachment microplates and grown using the liquid overlay technique^13^ as described previously until reaching a diameter of 450–550 μm.^3^ Spheroids were treated for 6 days with radioactive nutritional medium containing [^177^Lu]­LuCl_3_ or [^67^Cu]­Cl_2_ at *initial activity concentrations* (IAC) increasing from 0.125 to 1.25 MBq/mL.^3^ In one additional experiment on the effects of SSTR2-targeted RNT, spheroids were treated with the SSTR2 radioligand [^177^Lu]­Lu-DOTA-TATE for 2 h. Spheroids maintained in nonradioactive medium served as untreated controls. Treatments were terminated by partially replacing the radioactive medium three times with nonradioactive medium, each at a ratio of 1:3. This resulted in the removal of 96% of the radioactive medium. During subsequent follow-up, the medium was partially replaced every 2 to 4 days with fresh medium at a ratio of 1:2.^12^ In nine independent experiments, 5006 tumor spheroids were grown, treated, and monitored in individual cavities over a period of up to 35 days. During the experiments, imaging of the spheroids was performed with a bright-field microscope (Axiovert 40 CFL/AxioCam ICc1/AxioVision v4.8.2.0, Carl Zeiss, Oberkochen, Germany). The area around each spheroid was imaged and documented every 2–4 days after treatment start and for up to 35 days. Exemplary changes in spheroid morphology over the course of 35 days after radiation treatment are shown in Figure 1.

**Figure 1:**
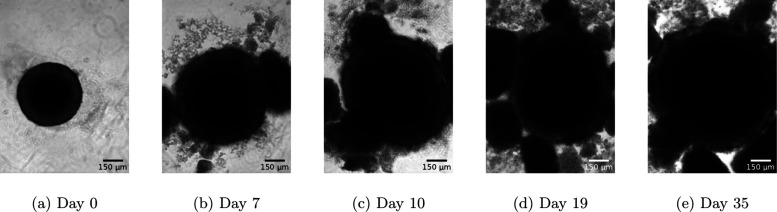
Visualization of a typical tumor spheroid progression with increasing amounts of treatment-induced debris and fragments imaged over a period of 35 days post radiation treatment.

### Ground Truth Definition

All 58380 images of spheroids, acquired within the above-mentioned nine experiments, were considered for inclusion in the present study. In these experiments spheroid delineation was performed with a threshold based method and was manually corrected if this deemed necessary as described previously.^12^ In the current investigation these delineations served as ground truth (GT) for network training. GT was reviewed and checked for suitability for network training. Due to the very large number of images not all data could be manually reviewed. Instead, for each plate only two to four spheroid images were randomly selected and visually inspected. If one of these images showed at least one of the following exclusion criteria, the whole plate was excluded. The reasons for exclusion were

(a) *empty GT:* no spheroid but only spheroid fragments were visible,
(b) *ambiguous GT:* data were identified as not manually corrected,
(c) *incomplete GT:* very large spheroids exceeding the field of view of the microscope.

Ultimately, 38090 images could be included in the present study. Figure 2 shows some examples of excluded data. The most frequent reason for exclusion was a strong treatment effect, as shown in Figure 2a. From a biological perspective, these cases are highly interesting; however, they are not suitable for network training. This also applies to cases with little or no treatment effect (Figure 2c), which can result in rapid spheroid growth that fills the entire field of view. Note that treatment effects are typically comparable across all spheroids on a given plate, which justifies reviewing only a few images per plate, as mentioned above.

**Figure 2:**
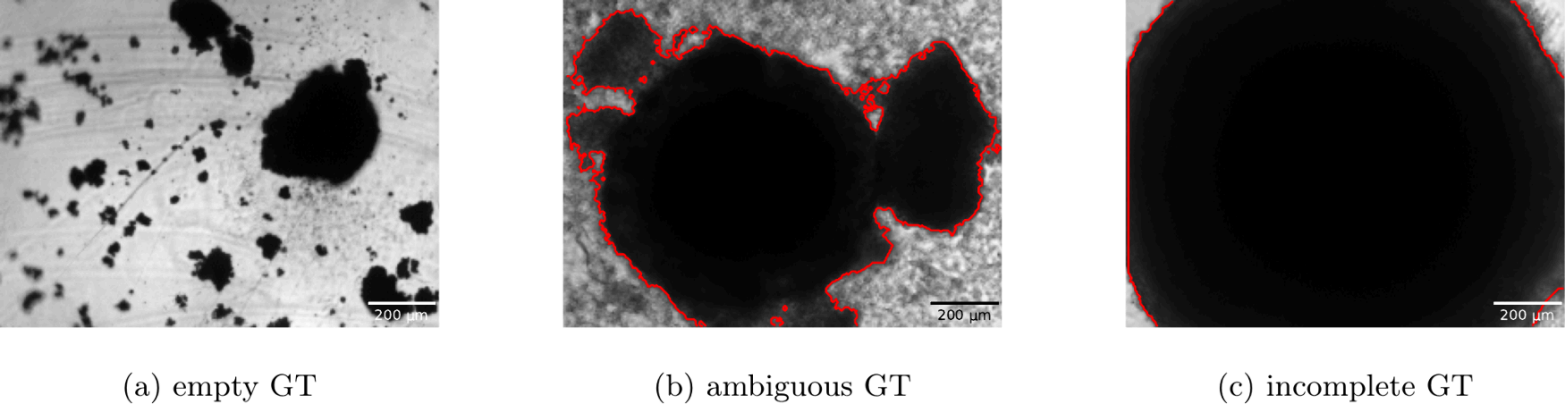
Examples of spheroid images that had to be excluded from training: Image (a) on the left does not contain a spheroid but only spheroid fragments. Image (b) in the center presents an ambiguous ground truth situation, making it unclear where the actual spheroid boundary should be located. Image (c) on the right does not fully cover the spheroid due to a too small field of view that hinders a sensible delineation.

The data were split into a main data set used for training and internal validation and a data set for independent testing using only data which were not included in network model generation. During splitting, all data from a single experiment were either assigned completely to the main data set or completely to the independent test data, resulting in N = 21567 and N = 16523 images in the respective data sets.

### Neural Network Training

The network training was performed using nnU-Net^11^ framework (version 2.5) based on PyTorch DL-library (version 2.3.1) for Python (version 3.10.14). It features a U-Net-like CNN architecture^7^ core and offers multiple on-the-fly data augmentations and self-configuration capabilities. The default planner was used for configuring the CNN given the training data set. Figure 3 illustrates the resulting CNN architecture. As shown, it is a deep U-Net-like CNN consisting of six resolution stages. The encoder part starts with 32 feature maps at a matrix size of 256 × 192; at each downsampling step, the number of feature maps is doubled while the matrix size is halved in each dimension, until a minimum size of 8 × 6 pixels is reached. The maximum number of the feature maps is capped at 512. The main building element of the U-Net is a convolution block comprising a convolution (kernel size 3 × 3), followed by instance normalization,^14^ and a leaky ReLU activation layer.^15^ Each encoder stage has two convolution blocks in a sequence; the first uses a strided convolution (stride 2 × 2) for the downsampling. The decoder has a similar structure to the encoder, but the feature maps are upsampled back to the original size using transposed convolutions (kernel size 3 × 3, stride 2 × 2). Skip connections^16^ between encoder and decoder are implemented via copy-and-concatenation of the feature maps at each resolution stage. Finally, the decoder output is projected via a 1 × 1 convolution into two feature maps containing unnormalized per-pixel class scores; normalization is then performed with a softmax function. The training followed the default nnU-Net procedure, namely using a SGD optimizer with decaying learning rate and deep supervision being employed. The batch size was set to 66.

**Figure 3:**
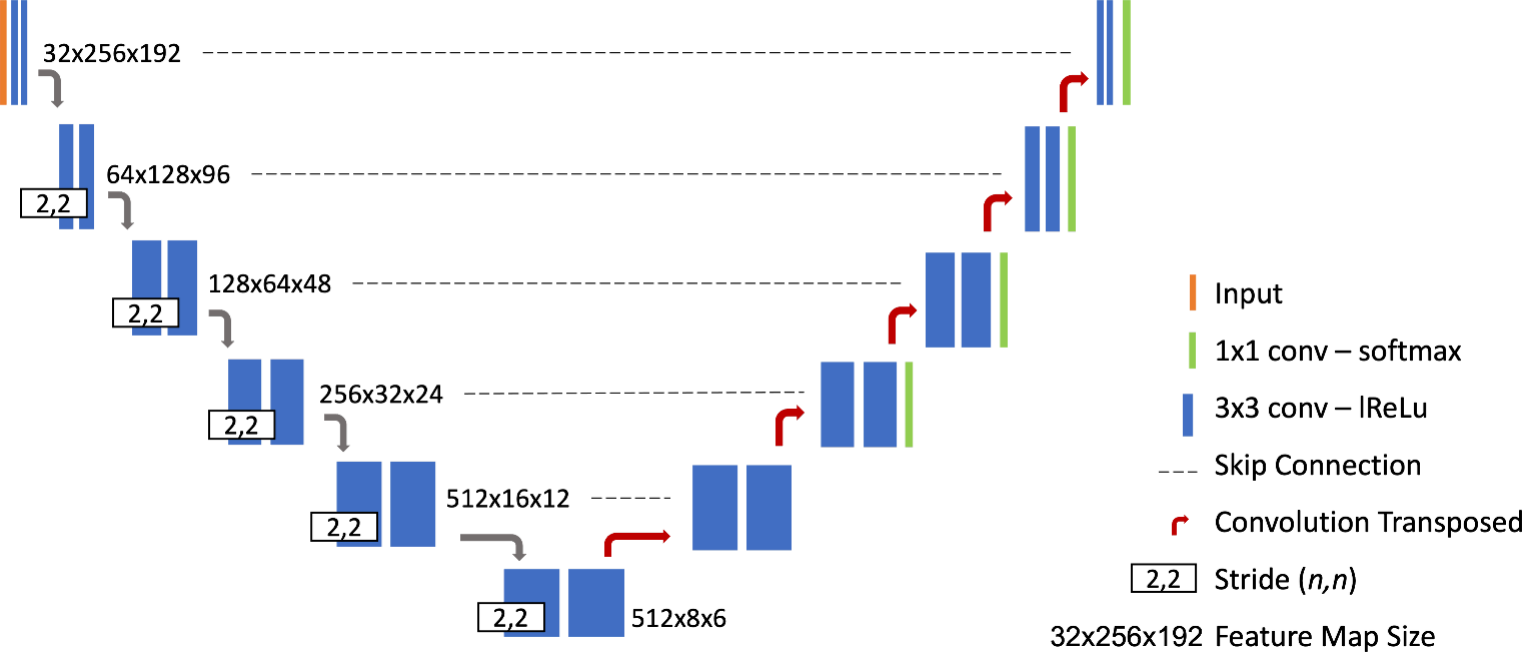
Illustration of the utilized U-Net–like architecture. The colored blocks represent operations, and the numbers beside them display the size of the tensors at each resolution stage (feature channels × spatial dimensions).

The training and inference were conducted on a system equipped with four NVIDIA Tesla V100S graphical processing units (GPU), each with 32 GiB of graphics memory. The system was running Ubuntu Linux 22.04.4 LTS and CUDA 12.2 with NVIDIA driver version 535.129.03 in place. Neural network training was performed using a 5-fold cross-validation scheme.^17^ For this, the training data set was split into 5 subsets and training was repeated 5 times using 4 out of 5 subsets for the network model development and the remaining (each time different) subset was held-out for consequent evaluation. The subsets were generated while ensuring a uniform representation of trials across them. Furthermore, all observations of each spheroid were ensured to be included in the same cross-validation subset to prevent cross-contamination throughout the different folds.

### Data Evaluation

For evaluation of the network performance in the training data set, each of the 5 models originating from the 5-fold cross-validation were used to predict the spheroid masks in the corresponding held-out evaluation subsets. These predictions were then pulled together to assess the network performance in the whole training data set. In the independent test data set, the predictions were performed with all 5 developed models in ensemble following the default nnU-Net procedure: first, the predicted pixel class probabilities were averaged across all five models and then binarized to produce the segmentation mask.

The spatial concordance between the network predictions and the GT delineations was quantified using the *Dice Similarity Coefficient* (DSC)^18^ defined as the double of the area of intersection between the delineations divided by the sum of their respective areas. The accuracy of the spheroid area determination by the CNN was assessed via computing the relative areas *S* = *A*_*cnn*_/*A*_*man*_, where *A*_*cnn*_ and *A*_*man*_ are the spheroid areas extracted from automated and manual delineations, respectively. Consequently, an *S* value of 1 indicates a perfect match between the predicted spheroid area and the GT area, and values greater than or less than 1 indicate the overestimation or underestimation of the spheroid area, respectively. Additionally, the comparison between *A*_*cnn*_ and *A*_*man*_ was presented as a scatter plot and a Pearson correlation analysis was performed.

Finally, we investigated the impact of the differences between manual and automated delineation on the half-maximum spheroid control dose (SCD_50_, the initial activity concentration resulting in a 50% loss of spheroid regrowth^3^). A full analysis of SCD_50_ in these data is the objective of ongoing studies. In the present investigation, we therefore only have exemplary considered the analysis of SCD_50_ in a single experiment to showcase the difference between a manual and CNN-based delineation.

### Generalization Assessment

To evaluate the generalization performance of the trained network, it was additionally applied to N = 7103 images from two independently conducted tumor spheroid experiments performed at our institute as part of ongoing investigations. Both experiments were conducted after network training and images were acquired using a different microscope (Axiovert 40 CFL/AxioCam 350/Zeiss ZEN v3.10, Carl Zeiss, Oberkochen, Germany) than the training data. Compared to the images used for training, N = 4800 spheroid images of radioresistant MPC cells (MPC-RR)^19^ and N = 2303 images of HepG2 tumor spheroids (HepG2-Red-FLuc; Bioware Brite; Revvity, part no. BW134280; RRID:CVCL_5I98) were included to further assess the network performance for the delineation of spheroids grown from different cell lines. Note that both experiments were not conducted specifically for evaluation of the automated spheroid delineation and employed different treatment protocols than those in the training data set. In brief, the additional MPC-RR cell lines were X-ray conditioned for several months prior to the experiment. This results in different growth rates, morphology, and fragmentation patterns in response to the treatment with doses between 0 and 40 Gy compared to the MPC spheroids used in the network training. In addition, in the HepG2 experiment, tumor spheroids were cultured under different conditions, including adipocyte-conditioned medium and resistin supplementation, to investigate their effects on spheroid growth and circularity.

All resulting CNN delineations in these additional data sets were visually inspected by experienced observers and manually corrected if necessary. This is the usual procedure of spheroid data processing at our site, independent of the delineation method. In the following, the manually corrected data were considered as GT. Network performance was assessed by comparing spheroid areas as well as the DSC of corrected versus uncorrected CNN-based delineations.

## Results and Discussion

The distributions of DSC characterizing the spatial concordance between manual and automated delineation in the cross-validation and independent test data are visualized in Figure 4. The corresponding statistics for the DSC distribution are given in Table 1. For the boxplots in Figure 4, statistical outliers were excluded to improve readability. The accompanying histograms include the full data (with frequencies capped at 500).

**Figure 4:**
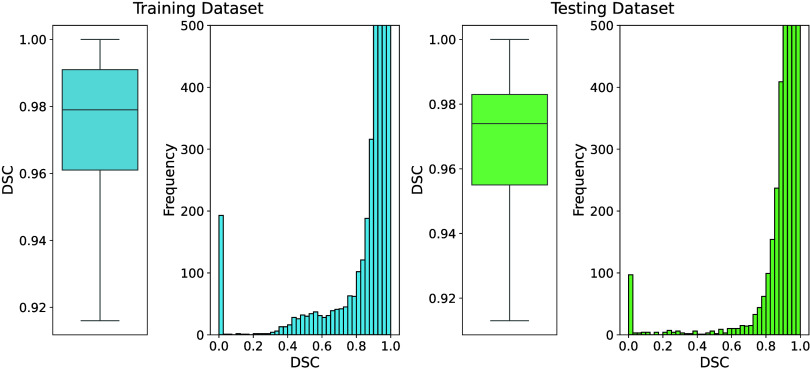
DSC scores between the GT and the network-generated delineations for the cross-validation part of the network training (left) and the independent test data sets (right). For better readability, statistical outliers were omitted from the boxplots, and histogram frequencies were capped at 500.

**Table 1:** Statistics for the DSC Distributions in the Training and Testing Datasets^a^

	DSC			N images		
Data set	Q_1_	Median	Q_3_	Total	DSC < 0.9	DSC = 0.0
Training	0.961	0.979	0.991	21567	1571 (7%)	189 (0.9%)
Testing	0.955	0.974	0.983	16523	1304 (8%)	90 (0.5%)

a Q_1_ and Q_3_ denote the first and third quartiles. Counts are shown for each data set, including the total number of images, and the number of cases with DSC < 0.9 and DSC = 0.0. Values in parentheses are percentages relative to the respective totals.

The boxplots indicate that the model yields a similar *median* DSC for the training data set (DSC = 0.979) and the independent test data set (DSC = 0.974). The interquartile ranges (*Q*_1_–*Q*_3_) reported in Table 1 suggest comparable variability across data sets: [0.961, 0.991] for training and [0.955, 0.983] for test data sets. Additionally, only 7% (training) and 8% (testing) of images yield a DSC < 0.9, reflecting poor concordance between the CNN delineations and the GT. Furthermore, 189 (0.9%) images in the training data set and 89 (0.5%) of the images in the test data set yield a DSC = 0, representing cases where CNN delineation failed completely.

Similar findings to the DSC analysis were obtained from the spheroid area comparison. Figure 5 shows the distribution of the automatically derived spheroid areas relative to the GT areas for the training and test data sets; results are summarized in Table 2. The median relative area was nearly identical for both data sets: 1.000 for the training data set and 0.992 for the independent test data set, suggesting unbiased area prediction by the CNN. The scatter plot in Figure 6 shows the comparison of the absolute spheroid areas of the network predictions with the GT for the independent test data set. The plot demonstrates a strong linear relationship (*R* ^2^ = 0.906). Points far from the line of identity represent cases where the network fails to correctly delineate the spheroid.

**Figure 5:**
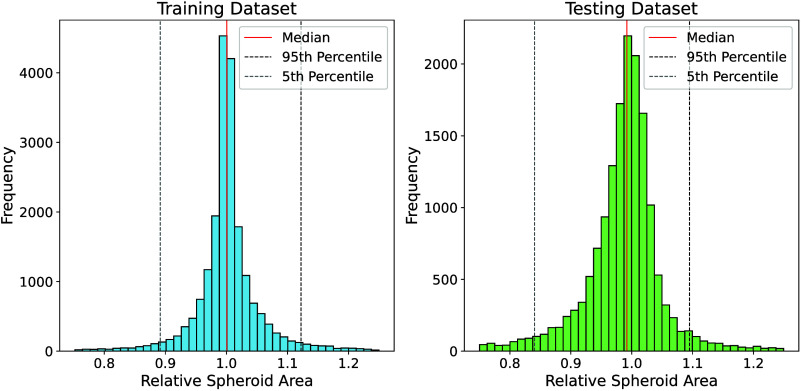
Histogram of the relative spheroid area for the training data set (left) and the test data set (right). For better visualization, only values between 0.75 and 1.25 are shown.

**Figure 6:**
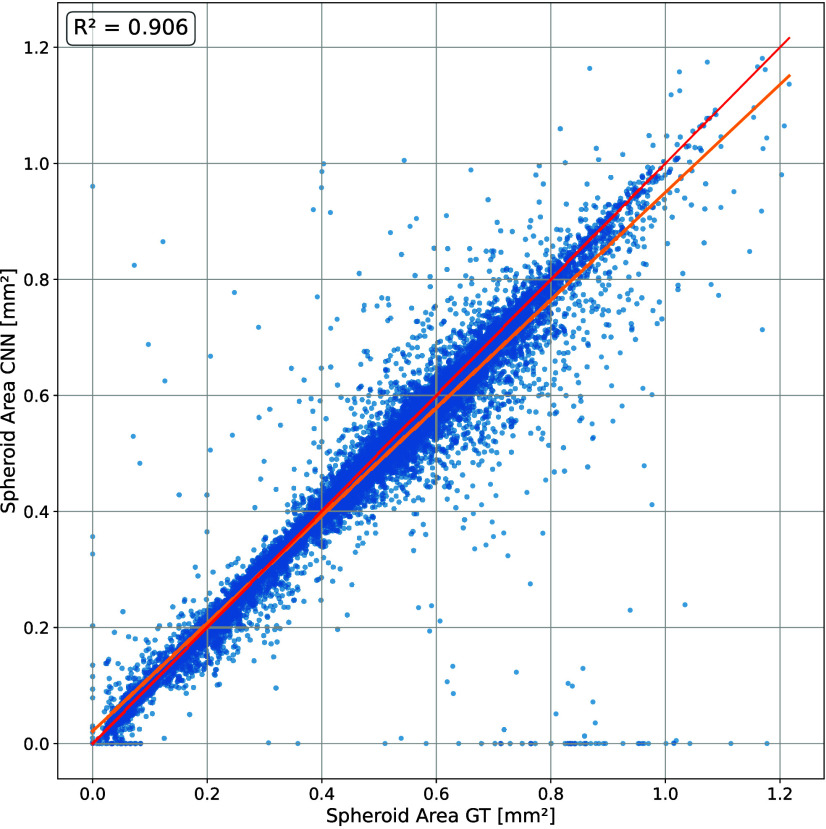
Scatter plot of spheroid area for ground truth (GT) versus CNN in the independent test data set. The red line indicates the line of identity (perfect agreement between CNN and GT), and the orange line indicates the regression line and a correlation coefficient of *R* ^2^ = 0.906.

**Table 2:** Distribution Statistics for Relative Spheroid Areas (*S*)

Data set	Mean	SD	Median	90% CI
Training	1.007	0.188	1.000	[0.891, 1.122]
Testing	0.988	0.204	0.992	[0.840, 1.094]

As outlined above, the model demonstrates satisfactory performance in the majority of cases, exhibiting a high overall DSC. The network achieves near-perfect delineations compared with the GT in approximately 90% of cases. In Figure 7, four examples of the network’s performance are presented. These examples illustrate typical scenarios with varying levels of difficulty, ranging from relatively straightforward cases to those complicated by surrounding debris. All examples show DSC > 0.9, indicating high concordance with the GT delineation.

**Figure 7:**
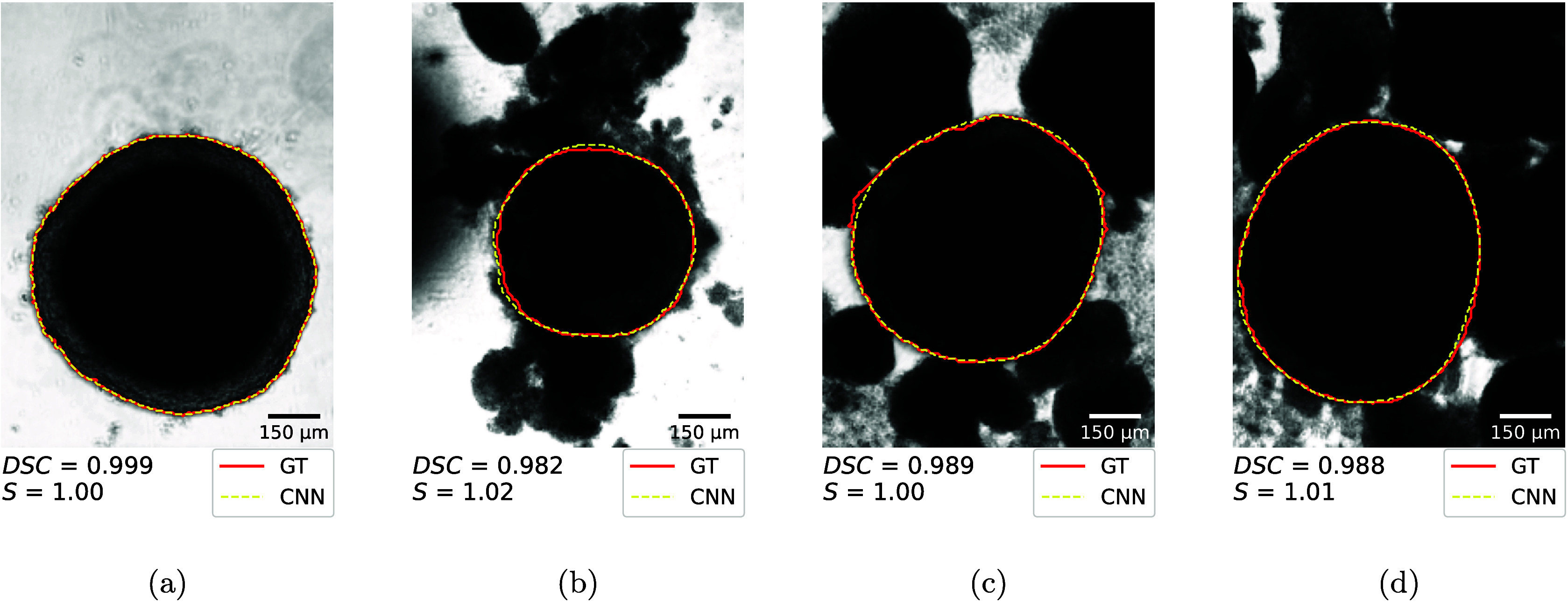
Exemplary images showing the performance of the CNN in comparison to the manual delineation at day 4 (a), day 8 (b), day 15 (c), and day 23 (d) for different spheroids. The red line denotes the ground truth (GT); the yellow dotted line shows the CNN prediction.

Figure 8 shows examples of typical cases where the CNN showed poor performance. We observed three main patterns of mismatch between the manually and automatically generated contours: First, the CNN delineates a different object than the one defined by the GT. This yields a low or zero DSC when there is little or no overlap (Figure 8a), which in fact for the DSC = 0.0 case only occurs in 0.9% and 0.5% of the cases in the training and independent test data set, respectively. Second, the model fails to capture the entire spheroid, resulting in only partial coverage (Figure 8b); this occurs in about 0.3% of the testing data and 1% of the main data set. Finally, the CNN sometimes fails to distinguish the target spheroid from nearby structures and oversegments, delineating multiple regions that could be misclassified as spheroids (Figure 8c). This occurs in about 0.4% of the main data set and 0.1% of the independent testing data set. It is worth noting, that in some challenging cases the GT itself might contain errors, thus a low DSC score does not immediately correspond to a poor CNN performance. This was actually the case in Figure 8a where the CNN identified the correct object as a spheroid while the manual GT was found to be actually wrong in that particular case. In summary, delineation failures can be attributed to two main situations: (1) images containing multiple circular structures, where the network sometimes delineates a different structure than the one annotated in the GT (Figure 8a) or all structures (Figure 8c); and (2) atypical contrast conditions (Figure 8b) that result in invalid delineations. Although we cannot fully explain why these constellations lead to delineation failures, we assume that such cases were underrepresented in the training data. However, this remains a hypothesis, as not all training cases were visually inspected.

**Figure 8:**
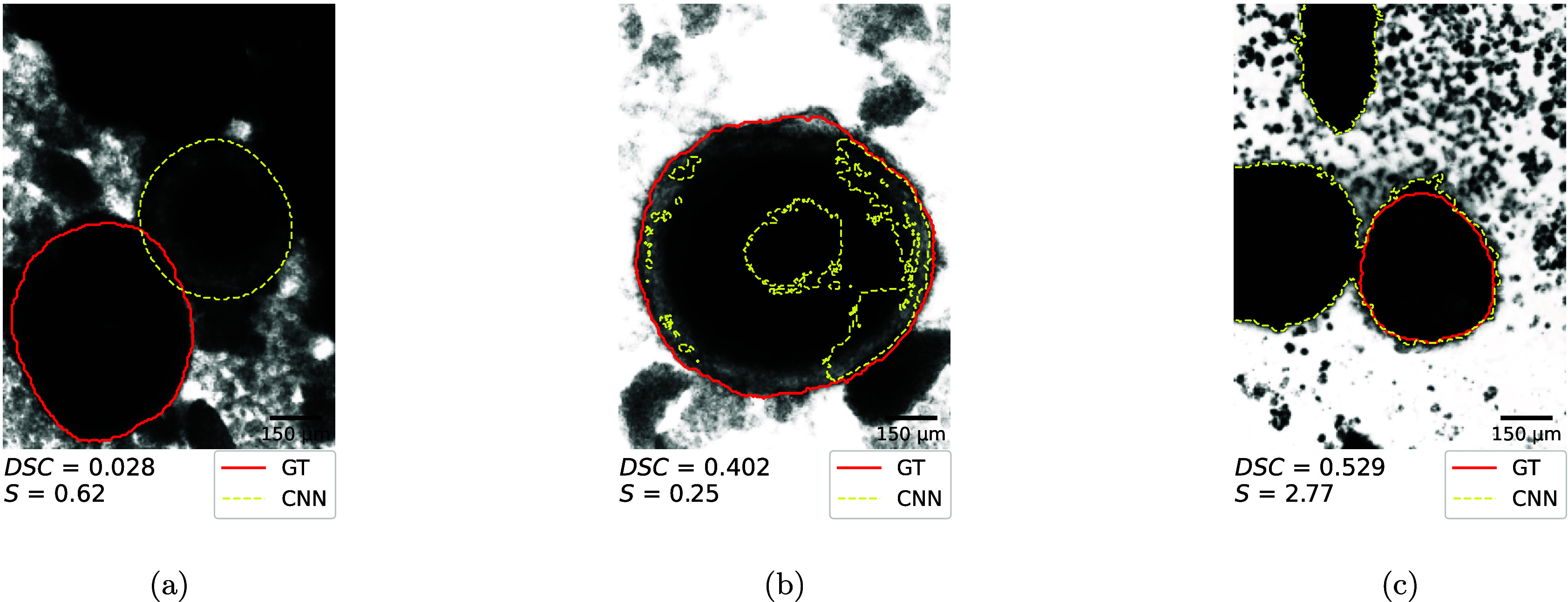
Exemplary images illustrating cases where manual and automated delineations mismatch. The cases include different (CNN is correct here) object selection (a), fragmentary delineation (b), and oversegmentation (c). The red line denotes the ground truth (GT); the yellow dotted line shows the CNN prediction.

The results of the spheroid growth analysis for an exemplary trial using manual and automated delineations are shown in Figure 9. The curves depict progression of the spheroid area over the course of the trial for *initial activity concentrations* (IAC) ranging from 0.00 to 0.40 MBq/mL measured over *N* = 96 spheroids for each IAC value. Across IACs, the trajectories are nearly identical. Minor deviations appear at lower IACs (0.00 – 0.05 MBq/mL) from day 27 onward, where the manual delineation yields slightly larger areas than the CNN; for moderate to higher IACs (0.10 – 0.40 MBq/mL), the manual and CNN curves are nearly indistinguishable.

**Figure 9:**
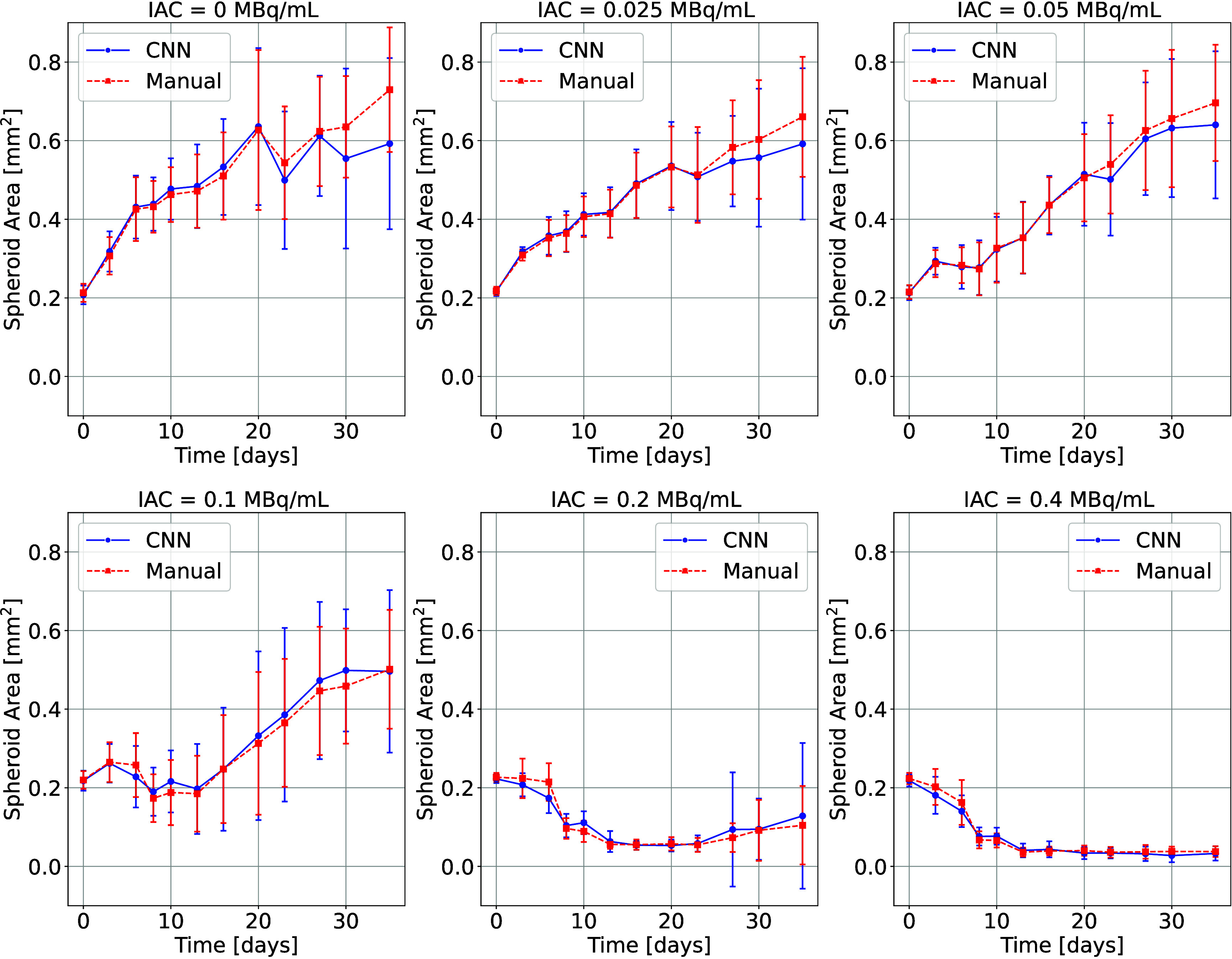
Comparison of spheroid area growth using manual (red) and CNN-based (blue) delineations. Shown is the spheroid area over the course of the trial (averaged over 96 spheroids) for different applied radioactivity concentrations; error bars indicate the standard deviation.

The corresponding dose–response curves in Figure 10 compare the spheroid control dose on two selected days for the manual (top) and CNN-based (bottom) delineations. Day 13 represents the middle of the experiment, while day 35 marks the late-experiment time point. For both days, the estimated control doses obtained with both delineation methods were nearly identical with SCD_50_ = 0.086 ± 0.001 MBq/mL (manual) and SCD_50_ = 0.083 ± 0.002 MBq/mL (CNN) for day 13, and SCD_50_ = 0.150 ± 0.001 MBq/mL (manual) and SCD_50_ = 0.149 ± 0.007 MBq/mL (CNN) for day 35.

**Figure 10:**
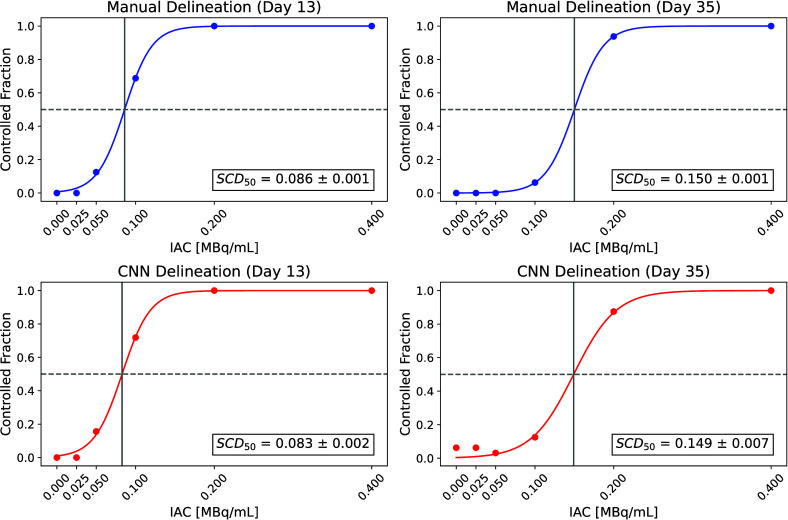
Comparison of the spheroid control dose for manual (top) and CNN-based (bottom) delineations on days 13 and 35. Plotted is the controlled fraction of spheroids for different initial activity concentrations (IAC), with the corresponding half-maximum spheroid control dose (SCD_50_).

It should be noted that the CNN-delineations were not manually postcorrected. The evaluation was performed with all above-mentioned delineation errors and still a very high agreement between the two delineation methods regarding SCD_50_ was achieved. This does of course not reflect the real world situation, where all data would be reviewed and corrected if necessary.

The manual corrections of the CNN-based delineations were, however, performed during the analysis of the generalization data set. Furthermore, a subgroup of these data (approximately 1000 images) was used to estimate the total time required for all steps, from applying the network to manual postprocessing of delineations, which resulted in approximately 2 h. This is about five times faster than the previously used method, which required roughly 10 h spread over several days for the same number of spheroid images. Note that the network itself requires only about one second per spheroid image to generate a delineation during inference. Thus, most of the time required for proper spheroid delineation was spent on visual inspection and sporadic manual correction of the spheroid delineations.

Network performance on the generalization data set is illustrated in Figure 11. Here, we again observed high agreement between the network delineations and the ground truth (which are, in this case, the same delineations but after manual correction). In only 851 cases (12%), the DSC was below 0.9, and the relative spheroid area was 1.032 ± 0.152 on average (90% CI: 0.957 – 1.349). Overall, 1555 of 7103 delineations (22%) required manual corrections. Furthermore, we observed a slight performance difference between the two investigated cell lines: the network tended to underestimate spheroid area for HepG2, whereas it showed a tendency to overestimate spheroid area for MPC-RR. Nevertheless, these results indicate that the network generalizes quite well.

**Figure 11:**
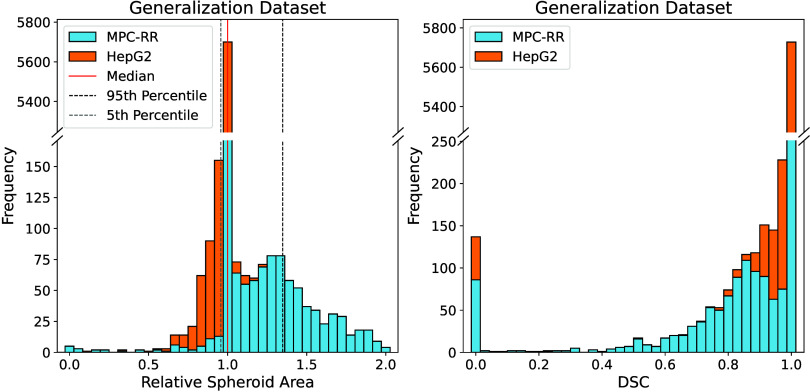
Histogram of relative spheroid areas (left) and DSC scores (right) for the generalization data set consisting of data from other experiments with different cell lines measured with a different microscope compared to the data set used for network training and testing. For improved visualization, only relative spheroid area values between 0.0 and 2.0 are shown. Note the discontinuous *y*-axis.

However, one limitation of our study still is that only data from a single microscope and cell-line were used for network training. Consequently, it cannot be ruled out that applying the method to other data may require retraining of the network with such data included, even though the results achieved in the generalization data set are very promising. Another limitation concerns the review of the ground truth. As noted above, it was not possible to review all spheroid images. Therefore, it is to be expected that the GT still contains delineations that would require manual correction. Nevertheless, the network’s overall performance suggests that this has only a minor impact, and incorporating additional corrected data for retraining will likely further improve delineation accuracy.

### User Interface – pyMarAI

To make the CNN-based delineation workflow more accessible, we embedded the entire procedure in a PyQt5-based^20^ graphical user interface called pyMarAI (Figure 12). It supports basic spheroid image data management, including automatic image-format conversion, and enables running delineation predictions on large spheroid data sets with support for GPU-accelerated computing environments. After delineation, the tool allows users to review images, tag delineation quality as “GOOD” or “BAD”, and can use the ROVER software (ABX GmbH, Radeberg, Germany) for manual corrections. Since August 2025, pyMarAI has been used routinely for spheroid delineation in all corresponding experiments. Additionally, the interface facilitates expansion of the training data set for potential retraining of the network by collecting all new and manually corrected delineations in a centralized directory structure. In the long term, this is expected to further improve the network’s delineation performance.

**Figure 12:**
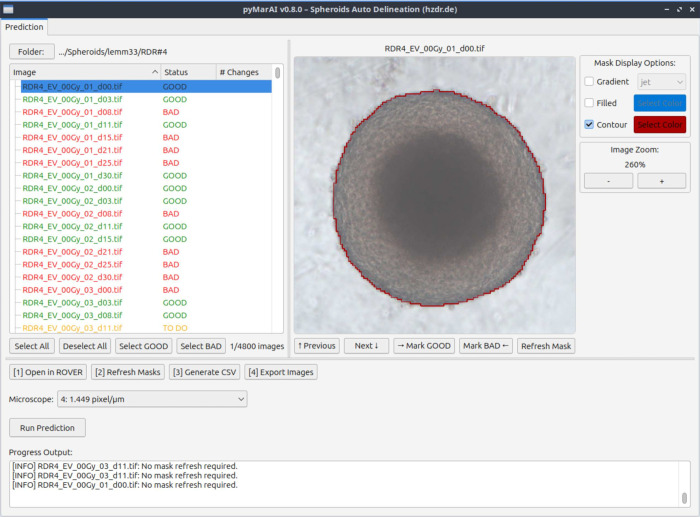
pyMarAI standalone application for automatic CNN-based spheroid delineation.

Finally, to enable interested institutions and third-party users to apply our network and potentially contribute to its further development, we have released the pyMarAI user interface and the corresponding trained model, along with detailed usage documentation under an open-source license.^21^

## Conclusions

Our network allows fast and accurate delineation of tumor spheroids in radiopharmacological treatment response assays, while rarely requiring more than minor manual corrections. This reduces the time needed for delineation of all spheroid images in a single experiment from several days with the previously applied method to a few hours only. It is planned to include more data in the training including data from different microscopes to further improve delineation accuracy and generalization. This will also further reduce the required time for manual corrections. The developed user interface helps to reduce the required processing time and also allows to easily feed back corrected delineations to perform network retraining steps to further improve the accuracy.
